# What Do We Know about Surface Proteins of Chicken Parasites *Eimeria*?

**DOI:** 10.3390/life13061295

**Published:** 2023-05-31

**Authors:** Jesica Daiana Britez, Anabel Elisa Rodriguez, Lucía Di Ciaccio, Virginia Marugán-Hernandez, Mariela Luján Tomazic

**Affiliations:** 1 Instituto de Patobiología Veterinaria, IPVET, INTA-CONICET, Nicolás Repetto y Los Reseros, Hurlingham 1686, Argentina; britez.jesica@inta.gob.ar (J.D.B.); diciaccio.lucia@inta.gob.ar (L.D.C.); 2 Instituto Nacional de Tecnología Agropecuaria, IPVET, INTA-CONICET, Nicolás Repetto y Los Reseros, Hurlingham 1686, Argentina; rodriguez.anabel@inta.gob.ar; 3 The Royal Veterinary College, University of London, Hawkshead Lane, London AL9 7TA, UK; 4 Cátedra de Biotecnología, Facultad de Farmacia y Bioquímica, Universidad de Buenos Aires, Junín 956, Ciudad Autónoma de Buenos Aires 1113, Argentina

**Keywords:** poultry, family poultry, coccidiosis, *Eimeria*, surface proteins, GPI, SAG, microneme proteins, vaccine candidates, One Health

## Abstract

Poultry is the first source of animal protein for human consumption. In a changing world, this sector is facing new challenges, such as a projected increase in demand, higher standards of food quality and safety, and reduction of environmental impact. Chicken coccidiosis is a highly widespread enteric disease caused by *Eimeria* spp. which causes significant economic losses to the poultry industry worldwide; however, the impact on family poultry holders or backyard production—which plays a key role in food security in small communities and involves mainly rural women—has been little explored. Coccidiosis disease is controlled by good husbandry measures, chemoprophylaxis, and/or live vaccination. The first live vaccines against chicken coccidiosis were developed in the 1950s; however, after more than seven decades, none has reached the market. Current limitations on their use have led to research in next-generation vaccines based on recombinant or live-vectored vaccines. Next-generation vaccines are required to control this complex parasitic disease, and for this purpose, protective antigens need to be identified. In this review, we have scrutinised surface proteins identified so far in *Eimeria* spp. affecting chickens. Most of these surface proteins are anchored to the parasite membrane by a glycosylphosphatidylinositol (GPI) molecule. The biosynthesis of GPIs, as well as the role of currently identified surface proteins and interest as vaccine candidates has been summarised. The potential role of surface proteins in drug resistance and immune escape and how these could limit the efficacy of control strategies was also discussed.

## 1. Introduction

*Eimeria* is a protozoan parasite genus that belongs to the phylum Apicomplexa. Most of the microorganisms in this phylum are zoonotic or cause an economic impact on livestock animals. Parasites of the genus *Eimeria* are closely related to other coccidian genera such as *Cyclospora*, *Toxoplasma*, *Neospora*, *Hammondia*, *Cystoisospora,* and *Sarcocystis*. They are more distant to *Cryptosporidium* and *Plasmodium* [1].

Chicken coccidiosis is caused by seven species of *Eimeria.* It is a highly widespread enteric disease, causing economic losses to the poultry industry worldwide. In 2016, the global impact was estimated at approximately €12 billion [2]. There are extensive studies focused on economic impact and epidemiology in the intensive poultry industry; however, little is known about small-scale productions (i.e., family household or backyard poultry). Household poultry production provides a valuable source of income in small communities and is recognised as an important contribution to food security and to decreasing rural poverty. According to the Food and Agriculture Organization (FAO) of the United Nations and a few reports from different part of the world [3,4,5], rural women are mainly dedicated to chicken husbandry and exposed to greater risks, but have little participation in decision-making and access to resources. However, when they market their productions, this activity constitutes an empowering tool for African women in rural communities [6,7]. Reports of coccidiosis in backyard productions stated a prevalence of *Eimeria* spp. ranging from 25.8 to 85.7% [8,9,10,11,12,13].

Species that affect chickens are *E. acervulina*, *E. brunetti*, *E. maxima*, *E. mitis*, *E. necatrix*, *E. praecox,* and *E. tenella*. Depending on the parasite species involved, infective doses, chicken immune status, and the breed line of the chickens, infections can vary from low to severe forms. Chicken *Eimeria* spp. that cause haemorrhagic disease are *E. brunetti*, *E. necatrix,* and *E. tenella*, and species that cause malabsorption are *E. acervulina*, *E. maxima*, *E. mitis,* and *E. praecox*. Broiler diets are formulated in accordance with the digestible and ideal amino acids (AA) concepts. There is an optimal ratio of AA to provide the exact balance needed for best performance and growth [14,15]. However, this ratio might change during a broiler’s lifespan due to age or physiological state [15]. Malabsorptive species alter the ideal AA profile due to biochemical and physiological changes in the intestinal epithelium, which impact the productive parameters (feed conversion, weight gain, or egg-laying). On the other hand, haemorrhagic species produce destruction of the intestinal mucosa, causing haemorrhages and severe diarrhoea that can lead to the death of the animal [2]. The most frequent species are *E. acervulina*, *E. maxima,* and *E. tenella* [12,13,16] but there may be differences between the type of production and geographical areas [13,17]. Infections with mixed species are common, but single infections have also been reported [12]. In addition to the seven chicken species, new cryptic species known as operational taxonomic units OTUx, OTUz, and OTUy have been recently described. They appeared to be geographically restricted, but new studies show a greater spatial distribution [17,18,19]. These OTUs have recently shown sufficient genetic and biological diversity to be considered new and distinct species and were named *E. lata*, *E. nagambie,* and *E. zaria*, respectively [20]. It is important to highlight that these new species could limit the effectiveness of current live vaccines.

Chicken coccidiosis is controlled by good husbandry measures, chemoprophylaxis, and/or live vaccination. The application of anticoccidial drugs usually added to food or drinking water has been used for more than sixty years and has led to the appearance of resistant strains for all *Eimeria* spp. over the world [21,22,23,24]. In addition, their use is under pressure worldwide, given the social influence in favour of the consumption of food free of chemicals, such as that produced in agroecological systems. Therefore, chemoprophylaxis faces new global regulations that limit or even ban its use. Live wild-type or attenuated vaccines have been effective in controlling the disease. However, wild-type vaccines can have safety issues and are not licensed for use in Europe, and attenuated vaccines possess a low reproductive index, increasing production costs, and limiting production capacity to meet worldwide demand [25]. To acquire full protection, chickens have to be vaccinated against each of the seven species of the parasite and in some cases with multiple strains, since they only confer specific immunity to homologous strains, making the manufacturing process highly demanding and stringent [26]. Therefore, the drawbacks inherent to live vaccination, and the emergence of resistant strains to traditional anticoccidials have raised concerns. The One Health paradigm has an integrated vision of health, considering animals, humans, and the environment. Antimicrobial resistance, food safety and security, and environmental contamination, among others, are issues concerning One Health; therefore, developing new cost-effective control strategies is crucial. This is especially relevant for small-scale poultry producers who have limited access to live vaccination given their high costs. Although the first live vaccines against chicken coccidiosis were developed in the 1950s [27], only two non-live vaccines have reached the market, these are Coxabic^®^ (Abic, Israel) [28] and Vac COX^®^ (Vetanco S.A) [29], this last one only commercially available in Argentina. One main barrier in protozoal vaccinology relates to the complex life cycles [30]. Furthermore, coccidiosis is caused by multiple species infections, and developing a universal vaccine that protects against heterologous species is not a simple goal to achieve. Effective vaccines against chicken coccidiosis may confer protection against multiple species, which differ according to the different types of flocks. Therefore, new multivalent designs should include protective proteins from—at least—the most frequent and pathogenic species to confer cross-protection. Numerous studies have demonstrated various levels of protection against vaccination with recombinant antigens, DNA, or live-vectored vaccines against coccidiosis. These results open the way to the pursuit of new prophylactic alternatives, for which the search for new candidates is paramount.

Surface proteins are of particular interest for studies on pathogen-host interactions as they are exposed on the outer membranes of parasites and establish interaction with host cells prior to invasion. They are exposed to the immune systems and could, therefore, be potential targets for developing protective immunity [31]. The rapid expansion of bioinformatics, genomics, transcriptomics, and immunoproteomics conducted in *Eimeria* spp. has increased the knowledge of protein function, fillings gaps in fundamental processes such as invasion and improving the first genomic annotations for these species [32]. All Apicomplexa contain in their genomes genes encoding for proteins localised on the surface of invasive stages, playing a key role in attachment, invasion, and elicitation of host immune responses. Antigens from the asexual reproduction stage, i.e., sporozoites, first generation schizonts and merozoites, have been related to immunoprotection against secondary infections [33,34]. In contrast, very few antigens from the sexual stages have been studied and be included in vaccine trials. From these stages, only GAM56 and GAM82, which have an important role in oocyst wall biogenesis and are components of the Coxabic^®^ vaccine, have been evaluated as vaccine candidates [35]. In the current review, we have compiled all the information regarding surface proteins of chicken *Eimeria* spp. The role of surface antigens in host immune evasion and drug resistance is also discussed, as well as the biosynthesis of the glycosylphosphatidylinositols (GPI) that anchors proteins to the parasite surface.

## 2. Life Cycle of *Eimeria* spp.

**Figure 1:**
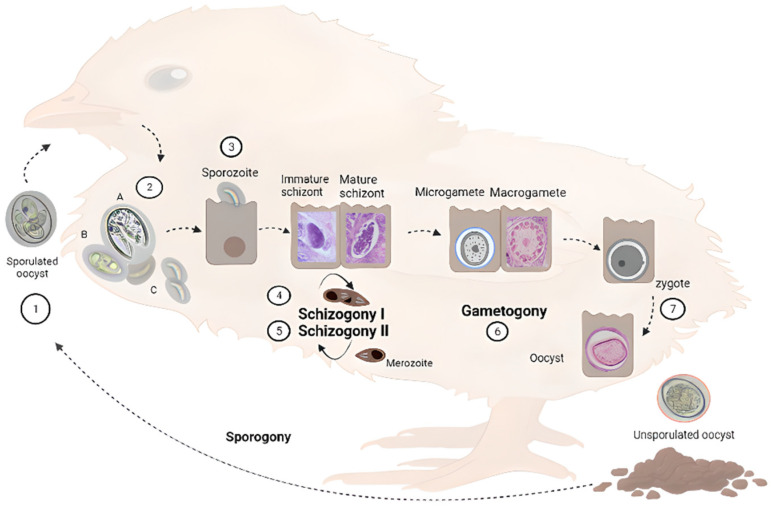
Schematic representation of *Eimeria* spp. life cycle in chickens. Parasites are transmitted among chickens via the faecal-oral route through water or food contaminated with sporulated oocysts (1). Upon ingestion, sporozoites are released in the gastrointestinal tract from sporocysts contained in the oocysts (2). Sporozoites invade intestinal epithelial cells—in different locations depending on the species—(3) and develop into the parasitophorous vacuole (PV) where they undergo schizogony (4), forming trophozoites that become immature and mature schizonts. After two to four rounds of schizogony (5), merozoites invade new cells undergoing gametogony (6). Zygote forms after fertilization of macrogametes by microgametes, which then develop into unsporulated oocysts (7) that are excreted with faces. In the environment, they undergo sporogony, restarting the life cycle. A, B and C are micrographs of freshly hatched parasites, i.e., oocysts, sporocysts and sporozoites, respectively, under a magnification of 400×. Immature and mature schizonts, macrogametes, and oocysts are micrographs of histological sections of intestines of experimentally infected birds stained with haematoxylin and eosin at 1000× magnification.

The *Eimeria* life cycle has been reviewed before [36]. Nevertheless, a schematic representation is shown in Figure 1 to introduce readers to the different parasitic stages that are mentioned in the current review. Different *Eimeria* species have different intestinal predilection sites and pathogenicity. This obligated intracellular parasite develops in the intestine of a single animal species, e.g., chicken (monoxenous), and comprises an asexual and a sexual cycle referred to as schizogony and gametogony, respectively. In addition, the parasite encompasses an exogenous (environmental) phase, necessary to become infective, termed sporogony. Oocysts are excreted and spread in the environment in a non-sporulated (non-infective) form. Sporogony initiates in suitable conditions of oxygen, temperature and humidity. Four sporocysts develop inside, each containing two sporozoites.

Sporulated oocysts disseminate in the environment, contaminating food and/or water, allowing coccidiosis transition by the faecal-oral route. When infective oocysts are ingested by chickens, sporozoites (Figure 1, step 2) are released in the gastrointestinal tract in a process called excystation, invading the intestinal host cells.

**Figure 2:**
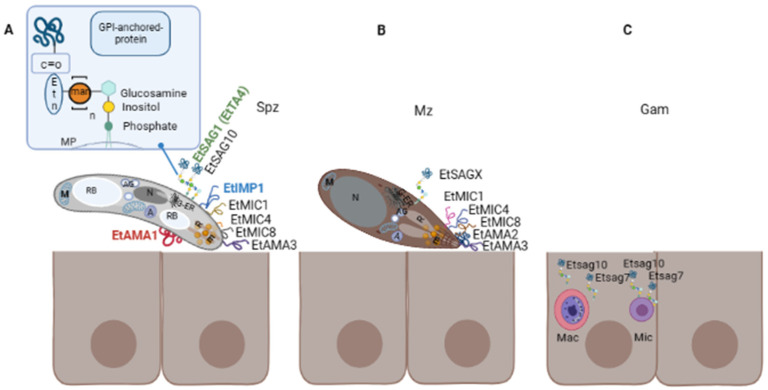
Surface proteins in three different life stages of *E*. *tenella* (Et). (**A**) Extracellular sporozoite possesses an apical complex composed of structural elements, rhoptries (R) and micronemes (m) that are involved in the invasion process, which comprises attachment, apical reorientation, formation of moving-junction (MJ) and formation of the PV. Eukaryotic organelles are depicted (nucleus (N), Golgi apparatus (G), endoplasmic reticulum (ER) mitochondria (M)), as well as organelles exclusive of the genus and the phylum or genus (refractile bodies (RB), apicoplast (A) and amylopectin granules (AG)). Studied surface proteins involved in these steps are EtSAG1 (TA4), EtIMP1, EtMIC4, EtMIC8, EtAMA1, EtAMA3, and EtSAG10. Proteins identified in other chicken species are indicated in different colours: green SAG1 identified in *E. acervulina* and *E. necatrix*; blue IMP1 identified in *E. maxima* and red AMA1 identified in *E. brunetti* and *E. maxima*. A representative scheme of the GPI anchor of SAG proteins is amplified in the upper right corner, this is composed of a molecule of glucosamine, n-mannoses (man), and an ethanolamine phosphate (etn). (**B**) Extracellular merozoite also possesses an apical complex. Identified surface proteins at this stage, including the first and second-generation merozoites, are EtSAG 2- 23 (referred to as EtSAGX), EtMIC1, EtMIC4, and EtMIC8 and EtAMA2. (**C**) Intracellular gametocytes. Transcriptomic analysis has identified two *sag* genes, *Etsag7* and *Etsag10*, which are upregulated at this sexual stage in *E. tenella,* they are represented in macrogametocytes (Mac) and microgametocytes (Mic); however, identification of the protein has not been reported.

Sporozoites possess classical eukaryotic organelles such as the nucleus, mitochondrion, endoplasmic reticulum (ER), and Golgi apparatus; they also contain typical structures of the phylum, i.e., conoid, apicoplast, micronemes, rhoptries, and refractile bodies (the last are specific of the genus) [37] (Figure 2A). The sporozoite cell surface is structured in a triple-layered pellicle, composed by the plasma membrane and the inner membrane complex, beneath, an array of sub-pellicular microtubules is found. In apicomplexan parasites, the host cell invasion process can be divided into attachment, apical re-orientation, formation of moving-junction structure (MJ), active invagination, and formation of the parasitophorous vacuole (PV) [37,38]. After invasion, intracellular sporozoites initiate schizogony, by growing inside the PV, forming trophozoites that later become multinucleated schizonts. Merozoites share characteristics of the sporozoites such as the triple-layered pellicle, nucleus, amylopectin granules, ER, Golgi, and apical complex, but they do not contain refractile bodies. Additionally, rod-shaped mitochondria and a vacuole are present in new merozoites [37].

The life cycle progresses with the formation of gametocytes that could become male and female gametes (micro and macrogametes) in a process termed gametogony. The diploid zygote is formed by the fertilization of haploid macrogametes by microgametes, which develops into an unsporulated oocyst. These oocysts are excreted with faeces and become infective in the environment, producing the consequent reinfection of chickens. The duration of the complete life cycle is variable, depending on the specific strains, usually lasting from five to seven days. Oocysts can persist in the environment for long periods, given their high resistance structure [36,39].

## 3. Surface Proteins of *Eimeria* spp.

Proteins located at the parasite surface are one of the first-acting proteins that are exposed to the host immune system, they recognise the host cell ligands and interact with them prior to invasion. In consequence, they could be interesting targets for immunoprophylaxis. In apicomplexan parasites, most of them are anchored to the parasite surface by a GPI anchor [40]. Importantly, GPIs of related parasites mediate strong immunomodulatory effects on the host immune system [41]. For example, recognition of GPI in malaria parasites by host Toll-like receptors 2 and/or 4, mediates a strong pro-inflammatory cytokine response from host cells in vitro [42]. Activation of the same receptors by *Toxoplasma gondii* GPIs seems to be critical to elicit an innate immune response [43]. Additionally, increasing evidence also suggests a role of GPI-proteins in host immune evasion in related parasites [44].

In the *Eimeria* genus, one of the most important and studied surfaces antigens are known as surface antigens anchored to the membrane by GPI (SAG), these are present in invasive stages (sporozoite and merozoite), interacting with the host before the invasion, and they are usually differentially expressed in different stages of the life cycle [45]. According to Reid et al., 2014 [32] there are 559 *sag* genes and 357 pseudogenes in the seven chicken species; *sag* genes are aggregated in three subfamilies: *sagA*, *sagB* and *sagC*. Whereas the former is present in all chicken species, *sagB* is only found in *E. tenella* and *E. necatrix*, with *sagC* restricted to the other five species. The three subfamilies possess a signal peptide at the N-terminal and a sequence for the GPI anchor at the C-terminal of the protein and an extracellular domain with six conserved cysteines, except for *sagC* which contains four instead [32]. They play an important role in the early recognition of the host, adhesion, and invasion as well as immune modulation [46,47].

In addition to SAG proteins, there are other proteins secreted from specialised organelles also involved in the host cell recognition and initiation of the invasion process: micronemes and rhoptries. They are present in the apical complex of Apicomplexa (Figure 2A) and all play essential biological functions for the invasion and development of the PV. Proteins secreted from micronemes (MICs) have been studied in different protozoan parasites, including *Eimeria* [48]. MICs secretion is upregulated through a calcium-mediated release pathway [49,50]. During the invasion, they display adhesive domains and are involved in gliding motility, migration, adhesion to the surface [51,52], and potentially in later egress from host cells after intracellular replication [53]. Some coccidian MICs harbour specific microneme adhesive repeat regions (MAR) domains that are involved in binding sialylated glycans [54]. It has been demonstrated that many of them, but not all, possess a transmembrane domain, among other relevant motifs, providing a dual localisation, i.e., in micronemes and surface membranes after secretion. This is the case for the *E. tenella* (Et) EtMIC1, EtMIC4 [52,55], and EtMIC8 [56], reasons why they are included in the current review. Apical Membrane Antigens (AMAs) are another well-known family of proteins secreted from micronemes and exported to the surface of the parasite. These were first identified in *Plasmodium knowlesi* [57] and *T. gondii,* which have been recognised as important vaccine candidates. Together with proteins from the neck of the rhoptries (RONs), AMA1 is involved in the formation of the moving junction (MJ) complex, a structure created between parasite and host to allow active parasite internalization and PV formation [58]. AMA1 from the apicomplexan parasites *Babesia*, *Toxoplasma*, *Neospora,* and *Plasmodium falciparum* has proven immunoprotective properties [50,59,60,61].

Immune-mapped protein-1 (IMP-1) is a highly conserved antigen located at the parasite surface. This was first identified in *E. maxima* [62] and later it was found in *E. tenella* [63] and other related Apicomplexa: *T. gondii* [64] and *N. caninum* [65]. Vaccination with IMP-1 induced a strong specific immune response [66,67].

Surface proteins studied for each chicken *Eimeria* spp. which have been considered therapeutic targets have been compiled in this section (Figure 2 and Table 1). No surface proteins have been described so far for *E. praecox*. Information related to constitutively expressed proteins of *Eimeria* spp. can be found in Olajide et al. [66].

### 3.1. Eimeria acervulina

Sporozoite and merozoite immunodominant proteins, with the ability to activate T- cells in vitro when expressed in *E. coli* fused to β-galactosidase, were identified in the early 90 s [67,68]. They were termed: cSZ-1, which encoded for a sporozoite surface antigen that appeared to be minority; cMZ-8, which was localised in a concentric pattern over the surface of merozoites, and whose recombinant form is recognised by sera from infected chicken; EAMZp30-47, containing a fragment in common in other surface and rhoptry proteins from *E. acervulina* merozoites; and MA1, a 22 kDa protein found in sporozoites. A few years later, Laurrent et al. [69] demonstrated that the protein cSZ1 initially identified by Jenkins et al. was a 19 kDa protein localised in the cytoplasm. The authors speculated that the iodination method used in the previous assays may alter the antigenic properties of the proteins; in consequence, the previous identification of these tentative surface antigens should be further confirmed.

Protein 3-1E is a profilin expressed at the surface of merozoites and sporozoites stages. This protein is highly conserved among *Eimeria* spp. [70,71] and plays a significant role in host-parasite interaction. Although profilins have usually a cytoplasmic location, the authors reported a surface localisation for *E. acervulina*. This superfamily of proteins prevents the polymerization of actin into filaments and, in certain circumstances, promotes actin polymerization. It has been demonstrated that a baculovirus-expressed recombinant 3-1E could induce the expression of IFN-ɣ in in vitro splenocytes stimulated with the recombinant protein, isolated from chickens challenged with *E. acervulina*; suggesting a cellular-mediated response. Immunoprotection against coccidiosis led by this antigen was also evidenced. Chickens vaccinated with a chimeric DNA vaccine, comprising cDNA encoding for 3-1E and mature chicken IL-15, have shown elevated levels of IFN-ɣ and IL-2 mRNAs [71,72]. These data indicate that Protein 3-1E is a suitable candidate antigen to be included in recombinant vaccines. Furthermore, this protein has been co-expressed with the Actin-depolymerizing factor, an actin-binding protein highly conserved in all eukaryotic cells; however, the immunoprotective properties of this combination have not been evaluated [73].

Few *mic* genes of *E. acervulina* (EaMIC2, EaMIC3, and EaMIC5) [56,74,75,76] have been identified and their function has been investigated. EaMIC3 has a key role in parasite survival since it inhibited cell apoptosis [74]. They all were found in the apical complex in sporozoites, but EaMIC2 and EaMIC5 are located diffused at both poles of merozoites. It was suggested that MICs might be translocated to the sporozoite surface when it comes in contact with the host cell [75]. The predicted transmembrane domain in EaMIC2 [77] overlapped with the signal peptide, suggesting that it is actually a hydrophobic region of the signal peptide rather than a transmembrane region. However, experimental confirmation is needed [51]. 

A proteomic analysis of *E. acervulina* sporozoites was conducted by Zhang et al., 2015 [78] in which chicken duodenal cells were co-cultured with soluble proteins of *E. acervulina* sporozoites. Upon electrophoresis, immunoblotting, and analysis by mass spectrometry, fifteen proteins were identified, including profilin 14-3-3, MIC, ROP, and a SAG (EAH_00011660). The results suggested that the identified proteins, including EaSAG, could bind to chicken duodenal, providing insight into the molecules and mechanisms involved in the parasite invasion process.

**Table 1:** Surface proteins in six out of the seven chicken *Eimeria* species.

Species	Protein	Life Stage	Tested as Vaccine Candidate	Function	References
*E. acervulina*	3-1E	Sz, Mz (A)	Yes	Host-parasite interaction	[70,72,79]
	SAG (TA4)	Sz (A)	No	Binding to duodenal cells	[78]
*E. brunetti*	AMA1	NR	Yes	NR	[80]
*E. maxima*	AMA1	Sz (A)	Yes	Host-parasite interaction	[62,81]
	IMP-1	Sz (A)	Yes	Invasion and immunoprotection	[62,82,83]
	SAG	Sz, Mz (A)	Yes	Host-parasite interaction	[84,85]
*E. mitis*	MIC3	Sz, Mz (A)	Yes	Host-parasite interaction, Immunoprotection	[86]
*E. necatrix*	NA4	NR	Yes	Immunoprotection	[87]
	SAGs (2)	Mz	No	Possibly attachment and host evasion	[88]
	3-1E	Sz, Mz (A)	Yes	Invasion, immunoprotection	[89]
*E. tenella*	SAG1 (TA4)	sOo, Sz (A)	Yes	Attachment, immunoprotection	[45,90,91]
	SAG	Sz (A)	Yes	Host-parasite interaction	[92]
	SAG2	Mz (A)	Yes	Elicitation humoral response	[92,93]
	SAG4	Mz (A)	Yes	Elicitation humoral response; cell-mediated immunity impairment, inflammatory response	[93,94]
	SAG5	Mz (A)	No	Elicitation humoral response, cell-mediated immunity impairment, inflammatory response	[93]
	SAG6	Mz (A)	Yes	Immunoprotection	[95]
	SAG7	Gam (S)	No	NR	[96]
	SAG8-9	Mz (A)	No	Host-parasite interaction	[93]
	SAG10	Sz, Mz, aSt (A) Gam (S)	No	Invasion, pathogenesis and immune evasion, drug resistance	[23,47]
	SAG12	Mz (A)	No	Elicitation humoral response, cell-mediated immunity impairment, inflammatory response	[93]
	SAG13	sOo, Sz, Mz (A)	Yes	Host-parasite interaction, possible drug resistance	[23,93]
	SAG15	Mz (A)	Yes	Elicitation humoral response, immunoprotection	[93,95]
	SAG17	Mz (A)	No	Host-parasite interaction	[45]
	SAG19	Mz (A)	No	Elicitation humoral response	[97]
	SAG23	Mz (A)	Yes	Elicitation humoral response	[45,93]
	AMA1	Sz (A)	Yes	Invasion (moving junction formation), Immunoprotection	[81,98]
	AMA2	Mz (A)	Yes	Not known, absence of immunoprotection	[81]
	AMA3	sOo, Sz (A)	No	Invasion	[99]
	IMP-1	Sz (A)	Yes	Immunogenicity and immunoprotection	[81]
	MIC1	Sz, Mz (A)	Yes	Association with EtMIC2 during attachment	[100]
	MIC4	Sz, Mz (A)	Yes	Adhesion, invasion, and immunogenicity	[100]
	MIC8	Sz, Mz (A)	Yes	Attachment, and immunogenicity	[56]

Abr. NR: not reported; Sz: sporozoite; Mz: merozoite; sOo: sporulated oocyst; Micgam: microgamont; Gam: gametocyte; A: asexual stage; S: sexual stage. To see previously identified 1 to 37 SAGs from *E. tenella* please refer to Tabarés et al., 2004 [45].

### 3.2. Eimeria brunetti

There are only two antigens reported for *E. brunetti*, EbMIC2 [101] and EbAMA1 [80] (Figure 2B). EbMIC2 has 77 and 71% similarity in their amino acids sequence with MIC2 of *E. maxima* and *E. tenella*, respectively. EbAMA1 was cloned and expressed in *E. coli* and its immunogenicity was evaluated in immunised chickens.

### 3.3. Eimeria maxima

An immunoprotective surface antigen has been recently identified through the construction of a cDNA expression library that was later screened through different rounds in an *E. maxima*-challenged model in chicken [84]. Upon the last round of immunisation, six individual clones of cDNA were identified corresponding to three hypothetical proteins, a rhomboid, a SAG (EmSAG), and a CAMP-dependent protein kinase regulatory subunit. An ORF of 708 bp encoded EmSAG, with a predicted molecular mass of 24.73 kDa and 13 predicted T-cell epitopes [84]. EmSAG proved to be an effective vaccine candidate against homologous challenges [85].

By using a combination of parasite genetics and selective barriers with population-based genetic fingerprinting, the protective antigens EmAMA1 and EmIMP1 were identified [62]. The former is involved in host-parasite interaction and is a single-pass type I membrane protein. EmIMP1 is recognised as a vaccine candidate and is highly conserved among apicomplexan parasites such as *N. caninum* and *T. gondii* [64]. *N. caninum* IMP1 may be involved in parasite invasion [65]. The C-terminal derivative of EmIMP1 could be used as a potent immunogenic candidate in the development of recombinant vaccines [82]. IMP1 transcripts were increased at the initial hours of sporulation between 6 and 12 h, and upon 18 h it was downregulated. Multiple sequence alignment has revealed seven single nucleotide polymorphisms, of which one of them could alter the IMP1 secondary structure. Additionally, indirect immunofluorescence with serum anti-IMP1 recognised *E. maxima* sporozoites but not merozoites, and immunohistochemical staining of *E. maxima*-infected chicken ileum tissue with specific sera, recognised intracellular parasite stages at up to 48 h post-infection. The authors suggest that these results may explain the immunoprotective effect previously observed [83].

### 3.4. Eimeria mitis

*E. mitis* is a frequent species found in backyard productions [13,102]. This species can enhance the pathogenicity of *E. tenella* and *E. necatrix* infections [103]. However, studies on this species are limited and only a few reports have included proteins from this species in vaccine formulation. One of the immunogenic antigens identified in this species was EmiMIC3 [86]. This protein is expressed on the surface of sporozoites and merozoites and more specifically in the anterior end of the sporozoites. EmiMIC3 possesses 9 MARs, with seven cysteine residues, in contrast to the 7 MARs of EtMIC3. The Type I MAR domain motif LxxY is present in MAR1, MAR2, MAR3, and MAR8 of EmiMIC3, as well as the coordinating binding motifs HxT [86]. It was suggested that the motifs in MARs of EmiMIC3 could contribute to site specificity during invasion and tissue tropisms. Immunogenicity of the native protein was demonstrated since it was recognised by rat polyclonal antibodies raised against the recombinant form (rEmiMIC3) produced in *E. coli.* Chicken immunised intramuscularly with rEmiMIC3 showed changes in T lymphocyte subpopulation, serum cytokines, and IgY levels, specifically increasing the proportions of CD4+ and CD8 + T lymphocytes, and the level of IFN-γ [86]; consequently, MIC3 from *E. mitis* was considered a good vaccine candidate to be included in multivalent vaccines.

### 3.5. Eimeria necatrix

A comparative transcriptomic study from merozoites performed by Su et al., 2017 [104], demonstrated that 2053 genes were differentially transcribed between *E. necatrix* second and third-generation merozoites (Mz-2 and Mz-3, respectively). From these, 99 transcripts corresponded to *sag* genes found to be differently expressed in Mz-2 and Mz-3. Sixty-four genes were upregulated only in Mz-3, which may indicate that they could be related to the different intestinal locations of both stages given that Mz-2 migrate from the mid-intestine to the caeca where they infect and develop into MZ-3. Additionally, an immunoproteomic analysis recently performed by Qu et al., 2022 [88] identified 50 immunogenic proteins from sporozoites, which were divided into nine groups according to their structure, function, and localisation. Group 9 comprises two SAGs antigens (ENH_00010300, and ENH_00078390), suggesting that they are good vaccine candidates as their *T. gondii* or eimerian counterparts. 

The antigen NA4 (Figure 2A) is a member of the sporozoite TA4 surface antigen family and showed 85% of identity with EtSAG1 (EtTA4, ETH_00010835) according to protein BLAST [105]. This antigen can induce a strong protective effect against *E. necatrix* [87].

### 3.6. Eimeria tenella

**Figure 3:**
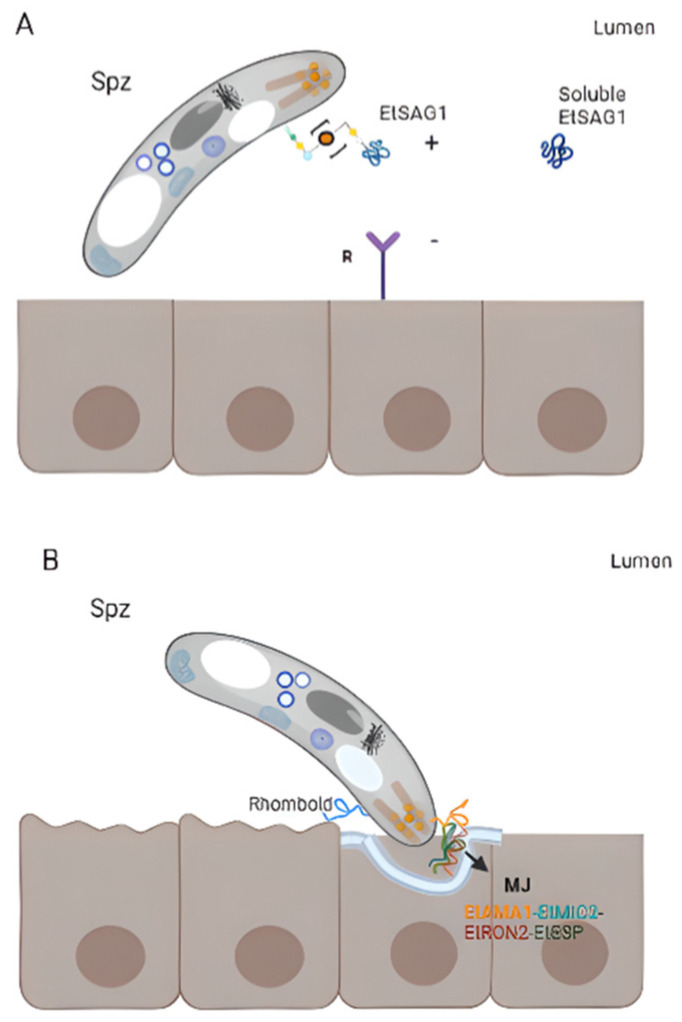

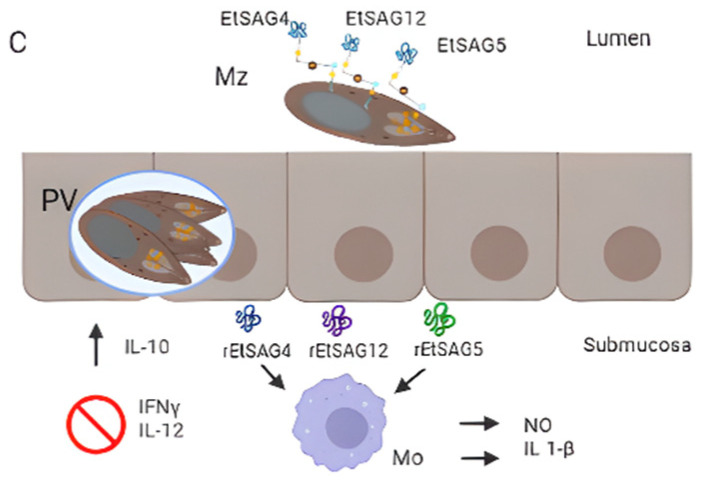
Surface antigens involved in host-parasite interaction. (**A**) Attachment. EtSAG1 at the surface of an extracellular sporozoite with a patch positively charged in the outward-facing (indicated with +), which might attach to negatively charged sulphated proteoglycans on the surface of the host cell (indicated with a −). The soluble form produced by the cleavage of EtSAG1 is also represented. (**B**) Formation of the moving junction (MJ) during invasion. EtAMA1 is represented in the apical region of a sporozoite (after being secreted by micronemes), interacting with an *Eimeria*-specific protein (EtESP), EtMIC2, and EtRON2 (grey). (**C**) Immunogenicity of EtSAG4, EtSAG5 and EtSAG12 in intra and extracellular merozoites. The recombinant forms of the three SAGs showed that they elicited pro-inflammatory cytokine IL-1b and nitric oxide (NO) produced by macrophages, inhibiting INF-ɣ and IL-12 and increased IL-10.

According to Reid et al., 2014 [32], *E. tenella* contains a total of 89 *sag* genes and 23 pseudogenes. One of the first and better-studied antigens of this species is TA4 also known as EtSAG1 (Figure 2A). This is a 25 kDa protein that is cleaved into 17 kDa and 8 kDa polypeptides, contains disulphide bonds, both transcript, and protein appear between 10- and 20-h post-sporulation, and the sequence contains conformational B- epitopes [106]. EtSAG1 can bind epithelial cells in vitro, playing an important role in parasite attachment to the host cell surface before invasion, and it can also induce protective immunity against homologous challenge (Figure 3A) [32,46,87]. EtSAG1 is localised on the surface of extracellular sporozoites as well as after invasion into human hepatocyte cells in in vitro cultures. Antigen shedding outside and inside the infected cells was suggested [46]. Interestingly, a positively charged patch in the outward-facing surface of EtSAG1 seems to attach to negatively charged sulphated proteoglycans on the surface of the host cell, initiating host invasion (Figure 3A) [46]; resembling *T. gondii* TgSAG1 role [107]. Recently, the genetic diversity of this antigen—along with EtMIC2—was analysed among 231 *E. tenella-* positive isolates from China and India, and compared with Korean isolates. The study demonstrated that this antigen is highly conserved since little variability was found among isolates [108]. *Etsag1* in these isolates was classified into four different haplotypes and showed limited amino acid polymorphism. Seven amino acid changes with low frequencies were found and, interestingly, amino acid changes identified in Korean and Chinese sequences were located in a putative B-cell epitope, suggesting the importance of determining the genetic heterogeneity and evolution of genes to design improved *Eimeria*-control strategies. Tabarés et al., 2004 [45] identified 37 EtSAGs, from which 23 were clustered in two multi-gen families: family *sagA* including EtSAGs 1–12, showing a mosaic pattern with conserved and variable regions; and family *sagB*, including EtSAGs 13–23, exhibiting a more biased pattern with variation predominantly in the N-terminal. Several *sags* are differentially expressed in sporozoites and merozoites. While EtSAG1 is expressed only in sporozoites, seven SAGs were found to be expressed in both stages, with the remaining 29 expressed only in merozoites, demonstrating a wide repertoire. In addition, surface localisation was demonstrated for EtSAG1, EtSAG4, and EtSAG13; GPI- anchoring was shown for EtSAG8, EtSAG17, and EtSAG23. Chow et al., 2011 [93] studied the inflammatory responses of eight SAGs by producing their recombinant forms and measuring inflammatory mediators in chicken macrophages. From them, EtSAG4, EtSAG5, and EtSAG12 induced high levels of nitric oxide and IL-1β. Moreover, the three recombinant forms have altered the expression of interleukins, suggesting a weakening of cellular-mediated immunity (suppression of IL-12 and IFN-γ, with elevation of IL-10), (Figure 3C) [93]. 

A study focusing on genes differentially expressed between stages of *E. tenella* has identified genes encoding for EtSAG1 (TA4), EtSAG13, and EtSAG23, in addition to genes encoding for MICs, ROPs, small heat-shock proteins, and calcium-dependent protein kinase ROPs. Whereas EtSAG23 was found in early sporulation and second-generation merozoites, EtSAG1 and EtSAG13 were expressed in sporulated oocysts and sporozoite stages [91]. Thirty-six immunogenic proteins were identified in second-generation merozoites, including three surface antigens: EtSAG2, EtSAG4, and EtSAG12 [109].

A transcriptomic analysis performed in *E. tenella* gametocytes (the sexual stages) has identified 863 genes that were upregulated in gametocytes compared with merozoites and sporozoites (asexual stages) [96]. Besides the abundance of transcripts encoding for hypothetical proteins containing PAN domains that are involved in adhesion, oocyst wall proteins, and a macrophage migration inhibitory factor were also found upregulated in gametocytes; transcripts encoding for SAG7 and SAG10 were also detected [96]. Authors identified genes encoding for oocysts wall proteins, subtilisin, and oxidoreductase on macrogametocytes and a microgamete-specific fusion protein, shedding light on the sexual biology of *E. tenella.* Another study found that EtSAG10 localised in the surface of sporozoites, merozoites, and the initial asexual stages (trophozoites, and immature schizonts), and importantly, in the PV membrane. Polyclonal antibodies anti-rEtSAG10 diminished the capacity of sporozoites to invade chicken embryo fibroblast (DF-1) cells in vitro. *Etsag10* gene was downregulated and contained from one to ten mutations in strains resistant to anticoccidial drugs (maduramicin and diclazuril) [47]. Thereby, EtSAG10 might also be involved in host cell invasion, pathogenesis, and immune evasion [47]. A recent study conducted by Xie et al., 2020 [23] has performed a comparative transcriptomic analysis of *E. tenella* strains sensitive and resistant to diclazuril and maduramicin. The study showed 1070 differentially expressed genes (DEG) involved in the peroxisome, biosynthesis of unsaturated fatty acids, and fatty acid metabolism, and some DEGs coded for surface antigens, which were downregulated in the two drug-resistant strains studied. Specifically, EtSAG10 and EtSAG13 are differentially expressed between drug-resistant and drug-sensitive strains, being downregulated in the two resistant strains. 

The tri-dimensional structure of EtSAG19 has been recently solved [97]. The authors have demonstrated that the protein fold is a three-layer αβα sandwich, which resembles the structure of the CAP superfamily, present in unrelated eukaryotes such as insects or plants. Members of this superfamily are involved in defence mechanisms such as antifungal [110] or systemic acquired resistance in plants [111] or in blocking cardiac and skeletal human ryanodine receptors in lizard venom [112]; accordingly, a role in modulating host-immune system in defence of the parasite life was suggested for EtSAG19. 

*E. tenella* EtAMA1 is secreted by sporozoites micronemes and is critical for host cell invasion by the formation of the MJ, in association with rhoptry neck protein 2 (EtRON2), EtMIC2 and *Eimeria*-specific protein (EtESP) [98,113]. The latter is a secreted protein and it has been demonstrated that antibodies anti-EtAMA1 or combined with its associated protein significantly impair sporozoite invasion (Figure 3B) [98,113]. In addition, authors showed that host transcripts involved in cell signalling, regulation of metabolic processes, and cytoskeletal reorganization were downregulated, whereas transcripts implied in cytoskeletal organization, cell migration, and movement, were upregulated upon the overexpression of EtAMA1 in the DF-1 cell line, providing relevant data related to the molecular mechanism of this protein during invasion. The paralog EtAMA2 has been identified in merozoites. In vitro assays demonstrated that it did not impair the parasite invasion and did not show a protective effect when used as a vaccinogen [81]. EtAMA3 was recently identified [98]. It is a type I integral membrane with cysteine residues in domains I and II, as other apicomplexan orthologues; however, an amino acid variance was observed in domain III. It is localised in the apical region of sporozoites and is involved in host cell invasion as AMA1.

Interestingly, *E. tenella* IMP1 homologue can elicit specific antibodies and IFN-Ɣ responses in chickens, conferring partial protection against homologous challenge [62].

Among the eight MIC proteins identified in *E. tenella* so far, three (EtMIC1, EtMIC4, and EtMIC8) contain a transmembrane domain [56,100] and proved to be crucial during parasite invasion. EtMIC8 is expressed in sporozoites and merozoites, it is involved in host cell attachment and confers moderate levels of immunoprotection [56]. The analysis of the coding sequence has revealed a signal peptide at the N-terminal, low-complexity fragments, four tandemly arranged EGF-like domains with an incomplete EGF-like domain and a transmembrane domain at the C-terminal.

## 4. GPI-Anchor Biosynthesis

Many studied GPI-anchored proteins in *Eimeria* spp. have demonstrated key roles in attachment, invasion, and elicitation of the immune response. However, little is known regarding their biosynthetic pathway. GPI-anchoring was demonstrated upon *E. tenella* sporozoite treatment with phospholipase C that shifted proteins from the detergent to the aqueous phase [45]. Additionally, the presence of a GPI-anchor in soluble proteins can re-route proteins to the parasite surface in *T. gondii* and *E. tenella* [114,115].

According to the postulated GPI biosynthetic pathway for *P. falciparum* [116,117], the first two steps of biosynthesis take place in the cytoplasmic side of the ER. First, UDP-N-acetylglucosamine (GlcNac) and phosphatidylinositol (PI) are linked to form GlcNAc-PI, catalysed by Phosphatidylinositol N-acetylglucosaminyltransferase (PIG) subunit A (PIG-A) and Glucose-6-phosphate isomerase, GPI-1. Removal of the N-acetyl group is catalysed by PIG- subunit L and yields GlcN-PI. The following step is catalysed by PIG- subunit W, which involves the addition of a fatty acid to the inositol ring of PI, resulting in GlcN-acyl-PI, which is translocated to the luminal ER side by a flippase. Three mannoses are then sequentially added to GlcN-acyl-PI (or GlcN-PI), catalysed by PIG-subunit M, PIG-subunit V, PIG- subunit B. The mannose donor substrate is a dolichol-phosphate-mannose (DPM), which is synthesised in the cytoplasmic side by dolichol-phosphate-mannosyl transferase subunit 1, giving rise to dolichol-phosphate and a GDP-mannose, and then transported to the luminal side across the membrane. Then, the PIG-subunit O catalyses the addition of ethanolamine (Etn) to the third mannose in which the binding of the protein to the GPI anchor occurs. A four mannose is added by a SMP3 and the binding of the protein is catalysed by a GPI-anchor transamidase, GPI8, plus GPI transamidase component GAA-1. Thus, the final structure of the GPI anchor in *Plasmodium* is composed of a molecule of GlcN-PI, four mannoses, and Etn-phosphate that is attached to the C- terminal of the protein. For *Babesia bovis* a similar structure was proposed but lacking two mannoses [118]. Liu et al., 2016 [40] performed a comparative genomic analysis of the human Apicomplexa parasite *Cyclospora cayetanensis*. They showed high similarity with *E. tenella* regarding metabolism, invasion, and genomic organization but not for GPI biosynthesis and N-glycosylation. Specifically, the authors stated that the enzymes involved in the GPI biosynthetic pathway in *E. tenella* and *C. cayetanensis* are present except for the mannosyltransferases PIG-V and PIG-B, which are involved in the addition of mannose residues. Thus, the number of molecules of mannoses in the GPI linker is yet unknown (Figure 2A).

## 5. Chicken Coccidiosis Vaccines

Currently, two types of vaccines are available in the market to control poultry coccidiosis: live vaccines—either wild-type or attenuated—and a subunit vaccine. Live vaccines are produced in chickens (since life cycle cannot be completed using in vitro systems) and have been demonstrated to be effective in controlling the disease. However, wild-type vaccines can have safety issues if distribution of the vaccine is uneven in the flocks, and they are not licensed for use in Europe. Attenuated vaccines, based in precocious strains, possess a lower reproductive index, meaning the need of more animal for vaccine production, significantly increasing their costs and limiting production capacity to meet worldwide demand [25]. To develop full immunoprotection, chickens have to be vaccinated against all the chicken species of the parasite and in some cases with multiple strains, since there is no cross-protection between them [26]. Coxabic^®^ (Abic, Israel) was the first available subunit vaccine against chicken coccidiosis, which is based on purified antigens from gametocytes of *E. maxima* [26]. Protection is achieved by the production and transfer of immunoglobulins to the hatchlings through immunising breeding hens.

Recently, the first recombinant vaccine, termed Biotech Vac COX^®^ (Vetanco S.A), has been launched to the market in Argentina. This vaccine contains an eimerian protein expressed in *Bacillus subtilis*, which upon fermentation is inactivated, encapsulated in methylcellulose, and administered orally. The vaccine reduced oocyst excretion of *E. acervulina*, *E. maxima,* and *E. tenella* by 35.5% and a significant increase in body weight gain was demonstrated [29].

Developing new cost-effective control strategies, especially for family household poultry producers with limited access to live vaccination given the high costs, is crucial. In recent years, many attempts to develop next-generations vaccines have been made and were well reviewed by Blake et al., 2017 [119].

## 6. Discussion

In the context of One Health, it is essential to reduce the use of antimicrobials, including anticoccidial drugs such as sulphonamides, still used in many parts of the world. Resistance to anticoccidial drugs has raised high concerns over the world, and novel control strategies to reduce coccidiosis impact are being investigated. For example, the use of phytochemicals—reviewed by El-Shall et al., 2022 [120]—has emerged as an important alternative to classical anticoccidials. Vaccines arise as the most cost-effective alternatives to the use of chemicals. The discovery of novel immunoprotective proteins is crucial for the development of next-generation vaccines. To design improved control strategies, it is necessary to understand the epidemiology of poultry coccidiosis, and recent data have documented the occurrence of cryptic species, *E. lata*, *E. nagambie*, and *E. zaria* [20], which could impair the effectiveness of the current anticoccidial vaccines. As an example, a molecular study conducted in Australia [13] that includes commercial and backyard productions, has reported differences in the distribution of *Eimeria* spp., with the most prevalent species being *E. mitis* in small-scale productions as well as the occurrence of cryptic species, indicating the need to understand the impact of coccidiosis and the relationship between large and small-scale productions.

In the present review, we addressed the latest findings regarding proteins localised at the surface of the parasite, to compile current knowledge of these key players in host invasion, a very complex process in apicomplexan parasites. Surface proteins from chicken *Eimeria* spp. That are involved in host-parasite interaction were summarised in Table 1. It can be observed that most of them have been assayed as vaccine candidates and that 3, 1, 3, 1, 3, and 30 proteins were identified in *E. acervulina*, *E. brunetti*, *E. maxima*, *E. mitis*, *E. necatrix*, and *E. tenella*, respectively, indicating that apart from the pathogenic *E. tenella* the remaining species are little studied. *E. maxima* and *E. acervulina* are species causing malabsorption, also widely studied since they are found with high frequency in the field. In this sense, several immunogenic antigens have been studied in these two species: lactate dehydrogenase [121], MIF [87], cSZ-JN1 [122], cSZ2 [122], EaMIC3 [74], EaMIC5 [75] in addition to EaMIC2, 3-1E, and SAG1 [77] (Table 1). In the case of *E. mitis*, only one immunogenic protein, EmiMIC3, has been characterised so far [86]. Another pathogenic species with limited research is *E. brunetti*, for which only EbMIC2 and EbAMA1 have been identified (Table 1) [101]. Considering that infection occurs mainly by mixed species and that they are not cross-protective, including antigens to reduce the impact of species such as *E. brunetti*—which could generate haemorrhagic diarrhoea—or *E. acervulina* and *E. mitis*—which have been shown high frequency in different poultry productions [13,123]- would improve the vaccine coverage and effectiveness.

It is important to note that not only surface proteins expressed in asexual stages are good vaccine candidates, but also proteins expressed in the sexual phase were able to elicit host immune response. This is the case for gametocyte antigens GAM56 and GAM82 in *E. maxima* [124,125] that, despite not having a role in the invasion, are essential for oocyst biogenesis. A transcriptomic analysis performed in *E. tenella* gametocytes [96] has identified transcripts that are upregulated in this stage. It is important to note that 60.4% of the total upregulated transcripts in gametocytes encoded for proteins annotated as hypothetical, highlighting the poor understanding of this stage in *E. tenella*. Among the highest abundant transcripts, several gametocyte proteins such as EtGAM56, EtGAM82, EtGAM22, EtGAM59, and EtGAM230 were detected. So far, only two *Etsag* genes were found to be transcribed in the gametocytes stage [96]. Blocking this stage of the life cycle is another interesting prophylactic strategy. If this process could be suppressed by immunisation, it would be possible to block the transmission of *Eimeria* by reducing oocyst formation and excretion. Therefore, more studies on surface proteins at these stages would provide more insight into sexual reproduction, and possibly novel vaccine candidates would be identified.

*Eimeria* SAG proteins are widely recognised as key intermediates in attachment for the adhesion of parasites to host cells during the invasion, supporting the first step of the invasion process along with other proteins such as MICs. Importantly, SAGs elicit host immune responses, and novel possible roles have been suggested in immune evasion or drug resistance [23,46,93,126]. Although significant progress has been made in the past years, most SAGs remain uncharacterised. For instance, from a total of 559 identified *sag* [32] in the seven chicken species, only 4.65% (26/559) have been reported so far. According to Reid et al., 2014 [32], there are 16, 105, 39, 172, 119, 19 and 89 genes belonging to the *sag* family for *E. acervulina, E. brunetti, E. maxima, E. mitis, E. necatrix, E. praecox,* and *E. tenella,* respectively, from which only 1, 1, 3 and 23 has been experimentally identified in *E. acervulina, E. maxima*, *E. necatrix* and *E. tenella*, respectively, with none of them described in the remaining three chicken species. GPI anchor has been experimentally demonstrated in *E. tenella* SAG proteins nearly twenty years ago; however, its biosynthesis and structure remain to be elucidated.

Resistance to anticoccidial drugs in *Eimeria* sp. has been reported worldwide [21,22,23,24]; nevertheless, the molecular mechanisms involved in the development of drug resistance are not well understood. The study performed by Xie and collaborators [47] has revealed that EtSAG10 and EtSAG13 are downregulated in the resistant strains studied and that EtSAG10 harbours from one to 10 mutations. Interestingly, it was found that EtSAG10 was localised in the PV membrane. Therefore, authors hypothesised that EtSAG10 might escape the host immune response to protect the parasite against the intracellular environment. Altogether, these data suggest that this protein could be involved in pathogenesis and immune evasion. However, the precise role of EtSAG10 in the development of drug resistance and immune escape needs to be elucidated. Additionally, a transcriptomic study has revealed that *etsag10* was upregulated in gametocytes; this indicates that EtSAG10 seems to be the only SAG protein found both in sexual and asexual stages (Table 1). Shen et al., 2014 [127] identified 13 proteins in *E. tenella* merozoites, including two proteins involved in invasion and several metabolic enzymes, that were altered under the effect of the anticoccidial diclazuril. The two altered proteins involved in the invasion were EtMIC2 and an actin-depolymerizing factor, suggesting that the drug impairs the host-cell attachment and invasion. However, further studies are needed to elucidate the molecular mechanisms involved in drug resistance.

One of the mechanisms used by *Plasmodium* for the evasion of human immune responses is the variation of antigen expression between stages, polymorphisms and parasite sequestration [128]. For instance, the circumsporozoite protein (CSP), which is the major surface protein on *Plasmodium* sporozoites, is involved in the evasion of the immune response [128]. It is interesting to highlight that the only licensed vaccine against malaria, Mosquirix, is a recombinant vaccine that co-expressed a fraction of CSP and the hepatitis B surface antigen in a yeast-expression system, assembled into virus-like particles [129,130]. In this regard, Su et al., 2017 [104] have suggested that the lower expressed *sags* found in second-generation merozoite could be associated with immune host scape, benefiting the migration of parasites to the caecum, and that *sags* expressed in third-generation merozoite would be involved in attachment to caecum epithelial cells.

Low antigenic diversity has been reported for genes encoding for *E. tenella* surface antigens such as IMP-1, AMA1, SAG1, and MIC2 [108,131,132], a desirable feature for their inclusion in vaccines formulations since a high variability could impair its effectivity in different regions. Low variability for a protein is usually related to an essential function. One possible hypothesis for the low antigenic variability of antigens in a related parasite was raised recently by Delbecq, 2022 [44], who has stated that the essential role of GPI-anchored proteins in *Babesia* in host-parasite interaction could counteract the selective pressure of the immune system over the antigenic variability, therefore these genes possess low variability. Consequently, rather than *Eimeria* spp. displaying proteins with high antigenic variability like the *Plasmodium* CSP, the parasite would express interchangeable proteins with the same roles and little antigenic variability each, such as SAGs, that could help to immune system evasion, as has been described for *T. gondii* [133]. Nevertheless, mechanisms of immune evasion as well as the functional characterization of most chicken *Eimeria* SAGs remain to be elucidated.

This collated information emphasises not only the importance of accurately identifying *Eimeria* species in the different type of chicken production systems (e.g., different flock sizes or breed lines), but also the need to include proteins from the different species in the vaccine design, in addition to *E. tenella* (for which identification and functional characterization of more antigens is still needed).

## 7. Conclusions

This work aimed to review the literature to provide a clear picture regarding what is known about surface proteins, which have a key role in the invasion process, and that are the first host-cell interacting proteins accessible to the immune system. Therefore, they could be vaccine candidates of interest. Currently, the lack of recombinant or live-vectored vaccines commercially available makes the identification of proteins that play important roles in the invasion, parasite transmission, and evasion of the host immune response of special interest for developing new immunoprophylactic strategies. Improved alternatives for control to reduce the economic impact of coccidiosis at different production scales are paramount. Implementation of affordable measures for control of this complex parasitic disease in family poultry is of special interest, given that most of them do not have the resources to apply live vaccination. This would especially benefit women farmers, who are mainly dedicated to chicken production.

Most of the reviewed studies focused on proteins of the asexual stages of *E. tenella*, studies in other chicken species being very limited. Understanding the role of key proteins in all chicken species is essential to comprehend the dynamics of this multi-species disease and to the design of multivalent vaccines. The most studied surface antigens are SAGs from the asexual stages, but in the past few years, some antigens from the sexual stage (gametocyte) have been identified. Targeting proteins from this stage would allow the interruption of the parasite transmission, in a similar way to the coccidicide drugs sulphonamides act [134]. Although genomic, transcriptomic and immunoproteomic analysis has aided in the identification of new surface proteins, many of them are still annotated as hypothetical; therefore, they remain uncharacterised. Identifying proteins involved in immune evasion and drug resistance, and revealing their underlying mechanisms would also help in the design of improved control strategies.

## Data Availability

Not applicable.
